# Quercetin and Epigallocatechin Gallate Induce *in Vitro* a Dose-Dependent Stiffening and Hyperpolarizing Effect on the Cell Membrane of Human Mononuclear Blood Cells

**DOI:** 10.3390/ijms13044839

**Published:** 2012-04-17

**Authors:** Denisa Margina, Mihaela Ilie, Daniela Gradinaru

**Affiliations:** 1 Biochemistry Department, Faculty of Pharmacy, Carol Davila University of Medicine and Pharmacy, 6, TraianVuia Street, Bucharest 020956, Romania; E-Mails: denisa.margina@gmail.com (D.M.); danielagrdnr@yahoo.com (D.G.); 2 Toxicology Department, Faculty of Pharmacy, Carol Davila University of Medicine and Pharmacy, 6, TraianVuia Street, Bucharest 020956, Romania

**Keywords:** membrane fluidity, transmembrane potential, chronic hyperglycemia, quercetin, epigallocatechin gallate, human, *in vitro*

## Abstract

The bioactivity of polyphenols is closely linked to their ability to interact with biological membranes. The study evaluates the *in vitro* effect of quercetin and epigallocatechin on the membrane anisotropy and transmembrane potential of peripheral blood mononuclear cells (PBMCs) isolated from 26 type 2 diabetes mellitus patients compared to 25 age matched controls. The *in vitro* assays were analyzed in correlation with the biochemical and inflammatory profile of the subjects and with insulin resistance parameters (HOMA-IR, plasma resistin) as well. For type 2 diabetes patients, the increase of HOMA-IR and resistin concentration was associated with a significant decrease of the PBMCs membrane anisotropy. The two tested polyphenols induced a dose-dependent hyperpolarizing effect and stiffening of the cell membranes for all tested subjects. Physiological levels of quercetin and epigallocatechin gallate had the tendency to normalize the PBMCs membrane anisotropy of the cells isolated from diabetes patients, bringing it to the level of cells isolated from normoglycemic ones. Epigallocatechin gallate induced higher effects compared to quercetin on the membranes isolated from subjects with higher cardiovascular risk. The decrease of membrane fluidity and the hyperpolarizing effect could explain the cardiovascular protective action of the tested compounds.

## 1. Introduction

Vegetable based diets are considered health-promoting; their beneficial effects are largely attributed to the rich polyphenol composition [1,2]. Numerous data support the antioxidant potential of the phenolic phytochemicals and many other disease-preventative characteristics such as antimicrobial properties, anti-cancer activity, cardiovascular-related effects (reduction of dyslipidemia and atherosclerosis, endothelial dysfunction and hypertension, platelet activation and thrombosis) and the capacity to modulate blood glucose [3,4]. Even if a large number of studies have identified cellular targets that could be involved in the health promoting actions of dietary plant polyphenols, there are still controversies regarding the actual molecular interactions of polyphenols with those targets [2].

The bioactivity of flavonoids is closely linked to their ability to interact with membranes, since many of these compounds generate a cell response even if they are not internalized [5,6]. Polyphenols could affect cell function by modifying plasma membrane structure and physical characteristics such as fluidity and electrical properties [2,7–11]. Epigallocatechin gallate (EGCG), for example, interacts with the lipid rafts, thus disturbing the downstream activation of signals involved in the allergic response and in cell proliferation of colon cancer cells [2,12].

Since polyphenol interaction with membrane structures represents a major issue in modulating cellular events, the present study evaluates the *in vitro* short term effect of quercetin (Q) and epigallocatechin gallate (EGCG) on the membrane fluidity and transmembrane potential of peripheral blood mononuclear cells (PBMCs) isolated from patients with type 2 diabetes mellitus (T2D) compared to normoglycemic subjects. These effects of Q and EGCG on membrane properties were correlated with the biochemical and inflammatory profile of the patients as well as the insulin resistance parameters, aiming to evaluate if these biophysical mechanisms could explain the beneficial roles of polyphenols in cardiovascular disease (CVD) associated with hyperglycemia. The results of this study show which categories of patients might particularly benefit from the effects of the tested compounds on their cardiovascular risk. These studies could also be connected to the present trend of the general population of consuming large amounts of polyphenolic supplements.

## 2. Results and Discussion

Since we were aiming at finding the types of patients that could benefit from the effects of the polyphenols, the analysis of the *in vitro* results was performed in relation with the fasting plasma glucose level (Gli) and also with the total cholesterol (TC) level. For this purpose, we divided the subjects either in two groups (depending only on Gli) or in four sub-groups (depending both on Gli and the TC level).

Preliminary experiments proved that the two fluorescent probes (TMA-DPH and DiBAC_4_(3)) do not interfere with each other, nor with the tested compounds (Q and EGCG), and that they can be used in double staining experiments. We also assayed the response of cells (PBMCs) isolated from healthy donor to different levels of exposure (10 μM, 50 μM and 100 μM) of each of the tested compounds. These results, correlated with literature data showing that the physiological levels of plasma Q and EGCG reached after polyphenol rich meals are about 2–10 μM [13,14]; this made us select the 10 μM concentration to be tested for the cell isolated from the selected subjects.

### 2.1. Biochemical and Inflammatory Profile of the Selected Patients

**Figure 1:**
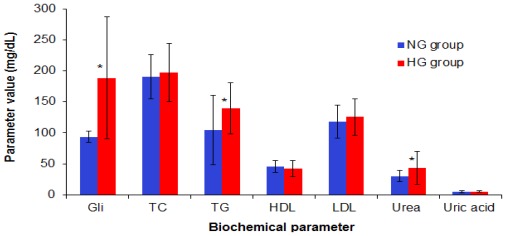
Biochemical characterization for HG and NG groups. Statistically significant different parameters are marked with an asterisc (*).

The glycated haemoglobin (HbA1c) was 8.15 ± 1.65% for the diabetes group (HG) and 5.35 ± 0.46% for the age-matched normoglicemia (NG) group (*p* < 0.01). Statistical analysis showed that there are significant differences between the diabetes patients and the normoglycemia subjects regarding the Gli, triglycerides (TG, *p* = 0.0108) and urea levels (*p* = 0.033). The significant differences are marked with an asterisc (*) in Figure 1. The uric acid levels (5.02 ± 1.52 mg/dL for NG group *vs*. 5.25 ± 1.44 mg/dL for HG group), as well as hepatic tests (AST—23.26 ± 9.95 IU (International Units) for NG *vs*. 22.00 ± 8.91 IU for HG and ALT—24.27 ± 20.03 IU for NG *vs*. 31.29 ± 20.32 IU for HG) had comparable values for the two groups.

The biochemical parameters for the subgroups, defined according both to their TC and Gli levels, are presented in Table 1. No significant differences were recorded between the 4 subgroups of patients for the triglycerides (TG) and high density lipoproteins (HDL) levels; the low density lipoproteins (LDL) level was significantly higher in the hypercholesterolemic (HC) subgroups compared to the age-matched normocholesterolemic (NC) subgroups.

The monocytechemoatractant protein-1 (MCP-1) level was significantly higher for diabetes patients compared to the normoglycemia group, but the intercellular adhesion molecule-1 (ICAM-1) levels were not different (Table 2). For C-reactive protein (CRP) level, the coefficient of variation was extremely high (90% for the normglycemia group and 129% for the diabetes group). As expected, the resistin and homeostatic model analysis for insulin resistance (HOMA-IR) are significantly different in the control and diabetes groups. The standard error values in cases of the HG group are extremely high, as the selected patients, even being all diagnosticated with T2D, were in different stages of the disease and were treated with different oral anti-hyperglycemic agents.

The inflammatory markers for the subgroups of patients are presented in Table 3.

The statistical analysis revaled higher levels of CRP in the HC subgroups compared to the NC ones; however, the differences were not statistically significant due to the high value of the standard deviations compared to the average for each group and subgroup. The ICAM-1 levels were not different between the study subgroups. The MCP-1 levels were significantly increased in hypercholesterolemia subgroups, compared to normocholesterolemia ones, for patients with normal levels as well as pathological levels of fasting plasma glucose.

Insulin was not statistically different between the four study subgroups. The resistin level and the HOMA-IR were significantly higher for HGNC and HGHC patients compared to the NGNC subgroup (Table 4).

### 2.2. Evaluation of Q and EGCG Effect on PBMCs Membrane Parameters

#### 2.2.1. Preliminary Experiments

**Figure 2:**
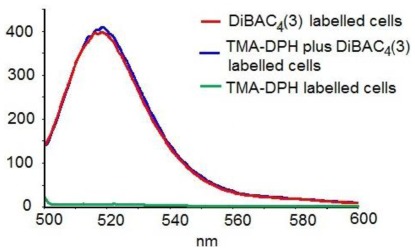
Fluorescence emission spectra of blood mononuclear cells (PBMCs) from a healthy donor, labeled with DiBAC_4_(3), TMA-DPH and DiBAC_4_(3) + TMA-DPH respectively (excitation at 493 nm).

Preliminary experiments proved that the maximum emission wavelength for the polyphenols is different from that of the fluorescent probes, so they do not interfere with each other. The data also demonstrated (personal results, in press) that the two probes do not interfere with each other and that they can be used in double staining experiments (Figure 2). As Q is dissolved in DMSO, we also checked the influence of the solvent on the cell membrane parameters (for PBMCs isolated from healthy subjects). The solvent induced only an effect of 0.28–0.30%, which can be considered negligible; therefore the changes in the cell membrane anisotropy were entirely attributable to quercetin.

#### 2.2.2. Evaluation of Q and EGCG Concentration Effect on PBMC Membrane Parameters

Data from the literature state that the physiological levels of plasma quercetin reached after polyphenol rich meals is about 2–10 μM, with 20–100 μM being considered a supraphysiological concentration [14]. We wanted to see if there were differences between the *in vitro* short term effects of both physiological and supraphysiological concentrations of the tested compounds on the biophysical parameters of the PBMCs membrane. For this purpose, we used cells isolated from two healthy donors, which were incubated for 20 min at room temperature with 10 μM, 50 μM and 100 μM of each of the tested compounds. The fluorescence intensity of DiBAC_4_(3) and TMA-DPH labeled cells were registered at baseline and after incubation with the tested polyphenols, in triplicate.

**Figure 3:**
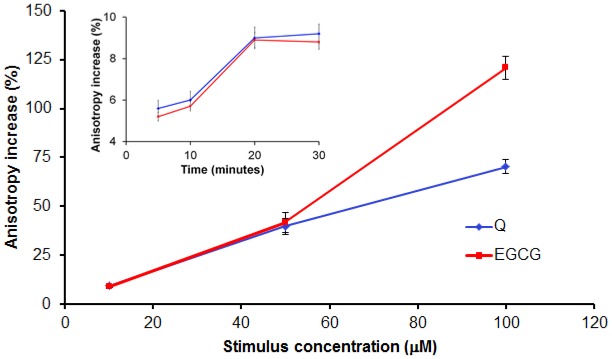
Q and EGCG induce a dose-dependent relative anisotropy increase (membrane stiffening) of PBMCs isolated from healthy donors. Insert presents preliminary data to set the incubation time.

In order to establish the time the polyphenols need to induce an effect on the cell membrane, we assayed the anisotropy and the transmembrane potential after 5 min, 10 min, 20 min and 30 min of incubation with the tested compounds. We observed that, at 20 min, the maximum effect was registered; the anisotropy and transmembrane potential did not change at longer incubation times (insert in Figure 3).

Both tested compounds lead to an increase of the membrane anisotropy, evaluated by means of TMA-DPH anisotropy; the increase of the stimuli concentration resulting in a higher anisotropy value (Figure 3), suggesting that increasing concentration of both polyphenols makes the cell membrane more rigid, as anisotropy and fluidity are inversely correlated [15].

**Figure 4:**
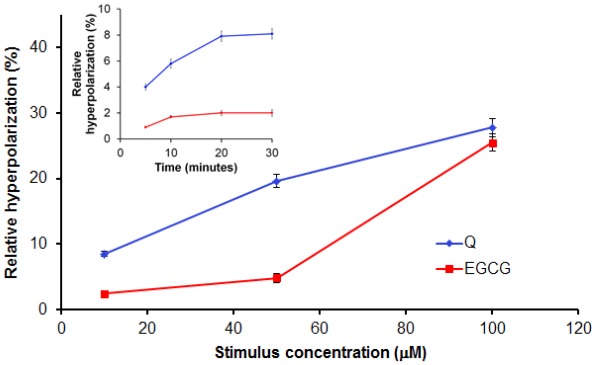
Dose-dependent relative hyperpolarizing effect of Q and EGCG on PBMCs isolated from healthy donors. Insert presents preliminary data to set the incubation time.

Q and EGCG also induced a dose-dependent membrane hyperpolarization, expressed by the decrease of the DiBAC_4_(3) fluorescence intensity in treated cells compared to those untreated (Figure 4).

#### 2.2.3. Effect of 10 μM of Q and EGCG on PBMCs Membrane Properties

The effect of 10 μM for each of the two stimuli was further evaluated on PBMCs isolated from all the selected patients, following 20 min incubation of the cells with the tested compounds. The effects of the tested compounds were first analyzed depending on the fasting plasma glucose of the patients.

**Figure 5:**
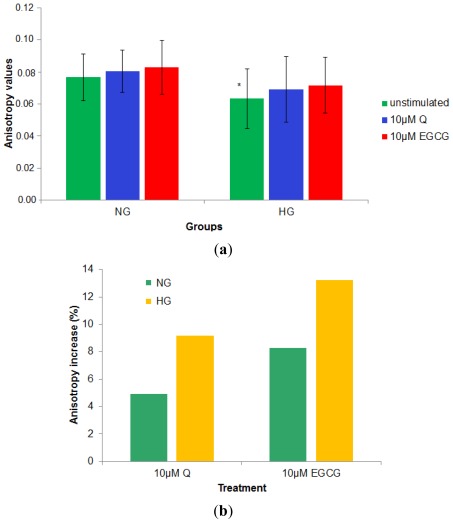
Q and EGCG effect on membrane anisotropy for NG and HG patients (**a**) absolute anisotropy values; (**b**) % increase of the membrane anisotropy (calculated according to Equation (2)) for the two groups under the effect of Q and EGCG (* denotes statistically significant differences between the baseline anisotropy values for HG group compared to NG group).

Baseline (unstimulated) PBMCs membrane anisotropy for the diabetes patients was significantly lower compared to the *r* value corresponding to the normoglycemia subjects, regardless of their TC level (0.0632 ± 0.0187 compared to 0.0766 ± 0.0147, *p* = 0.005). *In vitro* incubation of the cells with polyphenols led to an increase of the membrane anisotropy (stiffening of the cell membrane) in all cases. The effects were higher in the case of hyperglycemia patients for both compounds; also, EGCG induces a stronger increase of the membrane anisotropy in both groups, compared to Q. Physiological levels of Q and EGCG tend to normalize the PBMCs membrane anisotropy values of the cells isolated from hyperglycemia patients, making it similar to that of PBMCs isolated from controls (Figure 5).

**Figure 6:**
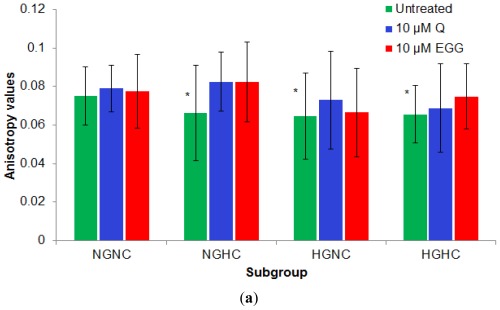

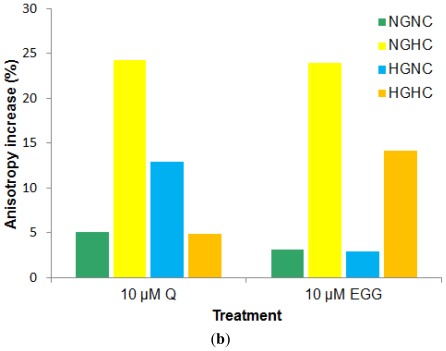
Q and EGCG effect on the PBMCs membrane anisotropy values in subgroups (**a**) absolute anisotropy value for each subgroup; (**b**) % membrane anisotropy increase (calculated according to Equation 2) under the effect of Q and EGCG (* *p* < 0.01, compared to NGNC subgroup).

The fluidity of a membrane is basically determined by the lipid composition of the cell membrane (phospholipid kind, phospholipids fatty acids composition and cholesterol content within the membrane) and the external temperature. Also, the membrane properties are directly influenced by their microenvironment [16–22]. As there is also evidence that membrane composition of immune cells is influenced by dietary intake of fatty acids [23], our experiments were performed on the same cell population exposed to the short-time action of polyphenols in comparison with untreated cells. Results were analyzed as a function of both Gli and TC values of the subjects, for the four subgroups of patients. The baseline values for the membrane anisotropy were higher for the cells isolated from normoglycemia-normocholesterolemia (NGNC) subjects, compared to the other three subgroups; values were significantly higher for NGNC subgroup compared to both HGNC (0.078603 ± 0.0126 *vs*. 0.063017 ± 0.0232, *p* = 0.033) and HGHC subgroups (0.078603 ± 0.0126 *vs*. 0.063389 ± 0.0148, *p* = 0.005). Patients affected by hypercholesterolemia, associated or not to hyperglycemia showed a reduced anisotropy value. The effect of the two tested compounds was the lowest on the more rigid membranes from the NGNC subgroup (0.2% effect for Q and 4.58% for EGCG). Q induced the highest effect on cells isolated from NGHC patients; EGCG had stronger effects on cells isolated from hypercholesterolemia patients, independent of the fasting plasma glucose levels. The membrane response to EGCG was higher in the subgroups with an increased inflammatory profile, (Figure 6). Thus, the increase of the anisotropy under the effect of EGCG was correlated (Sperman test) with the MCP-1 level, for the HGHC group (*p* = 0.038).

**Figure 7:**
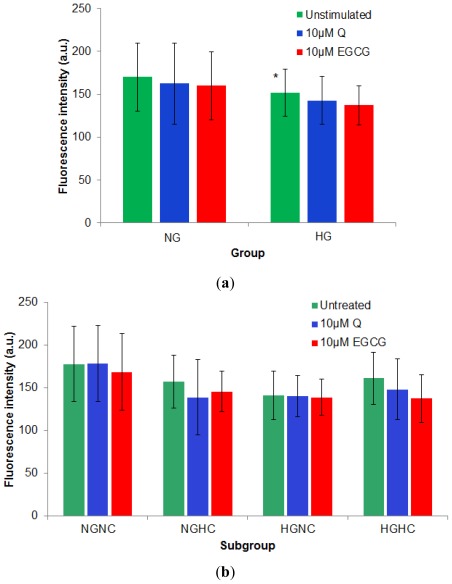
Hyperpolarizing effect (decrease in the DiBAC_4_(3) fluorescence intensity value) induced by treatment of PBMCs with 10 μM Q or EGCG. (**a**) for HG cells compared to NG ones; (**b**) for the subgroups defined depending on fasting plasma glucose and total cholesterol levels. (* denotes significantly reduced absolute fluorescence intensity of DiBAC_4_(3) labeled PBMCs isolated from HG group compared to the corresponding value from NG subjects).

Statistical analysis of the results examining transmembrane potential, reveal that the absolute fluorescence intensity of DiBAC_4_(3) labeled PBMCs isolated from hyperglycemia patients (diabetes group) regardless of their TC level is significantly reduced compared to the corresponding value from normoglycemia subjects (151.72 ± 27.11, compared to 170.03 ± 39.97, *p* = 0.05). Treatment of the cells with the two polyphenolic compounds leads to a reduction of fluorescence intensity of the DiBAC_4_(3) labeled PBMCs, corresponding to a hyperpolarization effect [24]. The EGCG hyperpolarizing effect was stronger for both groups (5.80% for normoglycemia subjects and 9.58% for diabetes compared to Q that induced a 4.42% effect on normoglycemia subjects and a 5.99% effect on HG patients). These results suggested a higher susceptibility of hyperglycemia to impair the voltage-gated activity of the ion channels, as membrane potential is mainly governed by the ions movement across the membrane and the hyperpolarization effect of the polyphenols was increased for HG cells. The NGNC cells had the highest fluorescence emission intensity value (10–20% higher than each of the other subgroups), while all the other three subgroups had similar values for this parameter (Figure 7).

#### 2.2.4. Discussion

Membrane anisotropy and transmembrane potential are among the most important biophysical properties of cell membranes. There are several research reports that link the microviscosity of cell membranes to the effects induced by different toxic stimuli. The increase of hepatocytes membrane fluidity under the influence of some xenobiotics (such as tacrine, ximelagatran or ethanol) is related to drug induced liver injury and leads to substantial disturbances of cell function [25,26]. Incubation with oxidized LDL particles induced an increase of the endothelial cells membrane fluidity and α-tocopherol prevented this effect [27,28].

According to literature data, flavonoids are modified in the small intestine and liver, mostly by deglycosylation. For example quercetin is metabolised and three major metabolites quercetin 3′-sulfate, quercetin 3-glucuronide and 3′-methylquercetin 3-glucuronide (isorhamnetin 3-glucuronide) are generated. Previous studies pointed out the fact that, at 10 μM level of exposure, the main metabolites of Q did not have a significant effect on the expression of inflammatory molecules, in endothelial as well as in smooth muscle cells [13,14]. In the case of the present study the metabolism of the tested compounds is not particularly relevant since the *in vitro* short-time exposure (20 min) to the polyphenols is tested, and we suppose that the differences in membrane properties are due to previous *in vivo* disturbances in the structure and/or functions of PBMCs from diabetes patients. Still, in correlation with literature data, we can assume the effects of the main metabolites of the tested compounds on membrane anisotropy to be minimum, since our results were analyzed in correlation with the inflammatory profile of the patients.

Literature data suggest several pathways for the role of dietary polyphenols: interaction with macromolecules [29], hormesis and synergy [30,31], interaction with estrogen receptors (ER) [32,33]; all of which have been demonstrated to induce cytotoxicity in cancer cell lines (breast cancer, osteosarcoma, human cervix epitheloid carcinoma, *etc*.). In a recent study by Galluzzo *et al*., doses from 1 μM, and up to 100 μM, were shown to decrease the number of cells in the human cervix epitheloid carcinoma cell line (HeLa) that were ER-α-positive but not the number of HeLa cells that were ER-α-delete [32]. Sotoca *et al*. recently proved that Q has increased affinity for ER-β at 50 αM [34]. According to Phiel *et al*. [35], peripheral blood CD8^+^ T cells and monocytes express low but comparable levels of both ERs. Also, Pierdominici *et al*. confirmed that CD4^+^ and CD8^+^ T lymphocytes, B lymphocytes and NK cells contain intracellular ER-α and ER-β [36].

In the case of our study, 47.06% were females (48% in the NG group and 48.15% in the HG group). Therefore we consider that the affinity of Q for estrogen receptors is not relevant, since the distribution of the patients depending on sex is quite similar between the two groups; so we considered that the effects on PBMC membrane fluidity and transmembrane potential are not greatly influenced by this factor.

We selected the membrane anisotropy of PBMCs as a marker of leukocyte function because literature states that the plasma membrane fluidity of these cells influences insulin resistance, insulin activity and also their phagocytic properties [16,37]. The literature regarding leukocyte membrane fluidity in diabetes is conflicting, probably due to the different methodology for cell isolation. Masuda *et al*. [38] reported a reduction of membrane fluidity for polymorphonuclear cells isolated from streptozotocin induced diabetic rats, Kantar *et al*. [39] showed that, in a group of type 1 diabetes patients the PMN membrane fluidity decreased compared to controls and Tong *et al*. [37] observed an increase in mononuclear membrane fluidity in a group of type 2 diabetics. Our results, in agreement with those obtained by Tong *et al*., showing the decrease of the membrane fluidity (increase of anisotropy) under the effect of the tested polyphenols, could illustrate a beneficial outcome of the flavonoid exposure; this conclusion is in agreement with several studies that link the increase of membrane fluidity for different types of cells with adverse/toxic effects of xenobiotics [25,26,37].

The evaluation of baseline membrane anisotropy for all the selected patients showed that diabetes patients had lower *r* values compared to the normoglycemia group, therefore the membranes of cells isolated from hyperglycemia patients, exposed to a mild inflammatory condition, are more fluid. Also, the decrease of the anisotropy value in diabetes patients, could be a result of the fact that hyperglycemia is associated with oxidative stress. The reactive oxygen species generated in excess in such conditions could influence in a negative manner the membrane function, thus having an impact on the membrane fluidity.

The adhesion of circulating leukocytes to endothelial cells plays an important role in the initiation of atherosclerosis. There are studies suggesting that elevated plasma levels of some cellular adhesion molecules (ICAM-1, VCAM-1, E-selectin) might be an index of endothelial activation and a molecular marker of early atherosclerosis [40]. Hyperglycemia, alone and associated with hypercholesterolemia induces an inflammatory response and an increased adherence of leucocytes to the endothelium.

Monocyte chemotactic protein-1 (MCP-1) is a chemokine expressed by adipocytes, smooth muscle and endothelial cells when exposed to inflammatory stimuli, which plays a crucial role in the recruitment of monocytes and T lymphocytes into tissues. MCP-1 level is stimulated by chronic hyperglycemia [41]. For the selected patients, the ICAM-1 level was higher for T2D patients, but the difference was not statistically significant; MCP-1 was the inflammatory marker that best discriminated T2D patients from normoglycemic ones. The association of hypecholesterolemia led to a significant increase of MCP-1 level for both normoglycemia and hyperglycemia patients. The MCP-1 level is significantly increased in hypercholesterolemia subgroups, compared to normocholesterolemia subgroups, for patients with normal (*p* = 0.003) and pathological levels (*p* = 0.03) of fasting plasma glucose. The patients characterized by both hyperglycemia and hypercholesterolemia were the most affected by inflammatory and cell adhesive impairments.

C-reactive protein is an extremely sensitive marker of chronic low-grade inflammation. Elevated high-sensitivity C-reactive protein (hsCRP) might potentially be a cause underlying the etiology and manifestation of type 2 diabetes, as well as being a powerful predictor of cardiovascular disease [42]. CRP distribution depending on both Gli and TC shows a large variation, but reveals the fact that the subgroup with high levels of both Gli and TC is characterized by a 84.51% increase of the hsCRP compared to the subgroup with normal values of both Gli and TC.

Human studies proved that resistin is expressed primarily in inflammatory cells and that may contribute to the metabolic and hemodynamic complications of obesity and hyperglycemia [43,44]. In our study, resistin levels increased significantly in HG patients, either NC or HC, the increase being correlated with that of HOMA-IR.

Our results show that the baseline membrane anisotropy had the highest value for cells isolated from normoglycemia-normocholesterolemia patients, compared to all other three subgroups. Patients affected by hyperglycemia, associated or not to hypercholesterolemia displayed a significant reduction of the anisotropy value. Not previously reported by literature, our results show that the *r* values are correlated with the resistin level and the HOMA-IR parameter for the patients; the significant increase of the resistin and HOMA-IR is associated with a decrease of the membrane anisotropy and therefore with an increase of the membrane fluidity.

Q and EGCG induced dose-dependent membrane stiffening in PBMCs isolated from healthy donors. The *in vitro* short term effect of 10 μM Q and EGCG resulted in an increase of the membrane anisotropy. The EGCG effect was higher on cells isolated both from normoglycemia patients and T2D patients. Our results could explain the beneficial effects of Q and EGCG supported by published literature, since previous studies have shown that the elevation of membrane anisotropy led to substantial disturbances of cell function and could be linked to toxic effects of different xenobiotics [26].

Analysis of the effects, depending both on fasting plasma glucose and cholesterol levels, shows that the effect of the two tested compounds was lower on the most rigid membranes from the NGNC subgroup. EGCG induced higher effects on the membranes isolated from patients characterized by hypercholesterolemia as well as by higher levels of MCP-1. Both polyphenols tend to increase membrane rigidity; Q and EGCG tend to normalize the PBMCs membrane anisotropy of the cells isolated from hyperglycemia patients, making it similar to that of PBMC isolated from controls.

The two polyphenols induced a hyperpolarizing effect on the PBMCs isolated from healthy donors, this effect also being dose dependent. Taking into account results of Verstraeten *et al*. [6] we can speculate that the effect on the transmembrane potential is due to the modulation of calcium fluxes.

Q and EGCG induced hyperpolarization in all patients. The effects were stronger in the case of cells isolated from hypercholesterolemia patients, with or without associated hyperglycemia, suggesting a higher defenselessness of the PBMCs to polyphenols in respect of the voltage-gated activity of the ion channels.

The reduction of the membrane anisotropy and transmembrane potential of PBMCs isolated from patients with hyperglycemia and/or hypercholesterolemia could be correlated with the increased risk of cardio-vascular disease indicated by literature data for these categories of patients. For subjects characterized by insulin resistance (increased levels of resistin and HOMA-IR), also reported as being with high risk of atherosclerosis, cell membranes were more fluid, compared to those of normoglycemia/normocholesterolemia; the decrease of the membrane anisotropy in these cathegories of patients could lead to an impaired function of blood cells and to an increased risk of stable interaction with the endothelial cells, constituting the initiating phase of atherosclerosis.

In direct correlation, the fact that the two tested polyphenols induced an increase of the membrane anisotropy for cells isolated from HG patients, bringing it to the level of that of cells isolated from normoglycemia subjects, could constitute a mechanism responsible for the reduction of cardio-vascular disease incidence reported for polyphenolic compounds. The effect of the tested polyphenols was higher for cells isolated from hypercholesterolemia patients, so in the case of these patients, the administration of polyphenol rich diets could have a higher impact on the cardio-vascular function, compared to the hyperglycemia patients [45].

## 3. Experimental Section

### 3.1. Materials and Devices

HPLC grade Q from Merck and EGCG from Sigma were used for the *in vitro* stimulation of the cells. The stock solutions (10 mM Q in DMSO and EGCG in water) were kept at −20 °C between different day experiments. Hystopaque 1077 from Sigma was used for the separation of PBMCs and RPMI 1640 (supplemented with l-glutamate) from Merck was used as the main medium for the cell suspensions during all tests performed. TMA-DPH (1-(4-trimethylammoniumphenyl)-6-phenyl-1,3, 5-hexatriene *p*-toluensulfonate) was purchased from Molecular Probes and used as a membrane anisotropy fluorescence probe. *bis*-(1,3-dibutylbarbituric acid) trimethine oxonol (DiBAC_4_(3)) from Molecular Probes was used as a fluorescent probe for the evaluation of transmembrane potential changes under the influence of the two polyphenols.

The biochemical evaluation of the subjects was performed using an Olympus 400 analyzer, with Biorad and Randox enzymatic commercial kits. The levels of hsCRP (C-reactive protein), MCP-1(monocytechemoatractant protein-1), ICAM-1 (intercellular adhesion molecule 1), insulin and resistin were evaluated on an automatic ChemWell 1000 system using ELISA kits (Invitrogen Inc.). The insulin resistance index (HOMA-IR) induced by homeostatic model analysis, was calculated depending on the fasting plasma insulin and fasting plasma glucose [46,47].

The cell membrane anisotropy and transmembrane cell potential evaluations were performed using a LS 50B Perkin Elmer spectrofluorimeter equipped with thermostated cell holder, magnetic stirring and fluorescence polarization accessory.

### 3.2. Study Design

We recruited 26 type 2 diabetes patients (HG group), 34 to 77 years old, diagnosed according to the American Diabetes Association criteria. Results were matched against a group made up of 25 normoglycemia subjects (NG group), 35 to 76 years old, who presented at the hospital for routine check-ups. Patients with severe renal, hepatic or hematological disease, overt cardiovascular disease or malignancy were excluded. All the patients in the study had a diabetes history of 2–4 years and were treated with metformin (46.15% of the HG group) or gliclazide (53.85%). None of the subjects had taken known antioxidants-containing supplements (vitamin C, vitamin E, probucol, *etc*.) two weeks prior to the study. All subjects in the normoglycemia group had a negative family history of diabetes. The study was approved by the local Ethics Committee and informed consent was obtained from the patients and healthy subjects.

For the selected patients, type 2 diabetes was diagnosed not only based on the fasting plasma glucose levels, but also on glycated hemoglobin. In the diabetes group we included patients with HbA1c levels higher than 6.6%.

We wanted to analyze data depending not only on fasting plasma glucose level, but also on the cholesterol level, so all selected patients were divided into four subgroups:

- NGNC subgroup, *n* = 15, included normoglycemia (fasting plasma glucose, Gli < 110 mg/dL) normocholesterolemia (total cholesterol, TC < 200 mg/dL) subjects.
- NGHC subgroup, *n* = 10, included normoglycemia-hypecholesterolemia (TC > 200 mg/dL) patients.
- HGNC subgroup, *n* = 12, included hyperglycemia (Gli > 110 mg/dL) normocholesterolemia patients.
- HGHC subgroup, *n* = 14, included hyperglycemia-hypercholesterolemia patients.

### 3.3. Biological Samples

Fasting venous blood samples drawn on Na_2_EDTA as anticoagulant were used for the isolation of PBMCs. The PBMCs were separated using the gradient density method, with Hystopaque 1077 [48]. Separated PBMCs (monocytes and lymphocytes) were washed three times with RPMI 1640 medium, standardized at 10^5^ cells/mL and used for the spectrofluorimetric assay [49,50]. Platelet rich plasma samples were used for the biochemical evaluation.

### 3.4. Methods

#### 3.4.1. Fluorescence Anisotropy Measurements

The cell membrane anisotropy measurements were performed by evaluation of TMA-DPH anisotropy values in a steady state fluorescence polarization experiment, after the cell membrane exterior phospholipids layer permeation of the probe [51–53].

The fluorescence anisotropy of TMA-DPH labeled PBMCs was evaluated prior and after 20 min of incubation, at room temperature, with Q or EGCG. For this purpose, the normalized cell suspension labeled with 2.5 μM TMA-DPH (2 min incubation, at 37°C, under continuous magnetic stirring) was excited with polarized light at 340 nm and the emission intensities were detected at 425 nm. The measurements were carried out for the four possible positions of the polarizers in the excitation and emission beam; each relative emission intensity value was computed as a mean of 200 points (measurements in a total time of 4 s in steps of 0.02 s). The calculation of the fluorescence anisotropy (*r*) was performed according to Equation (1):

(1) $$r=\frac{I_{vv}-G\cdot I_{vh}}{I_{vv}+2\cdot G\cdot I_{vh}}$$

where *I*_vv_, *I*_vh_, *I*_hv_ and *I*_hh_ represent the emission intensity when the polarizers in the excitation and emission beams are oriented vertical-vertical, vertical-horizontal, horizontal-vertical and horizontal-horizontal, respectively, and *G* = *I*_hv_/*I*_hh_ is an instrumental factor [54].

The effect was evaluated as relative anisotropy change following Equation (2):

(2) $$e_{r}=\frac{r_{f}-r_{i}}{r_{i}}\times 100$$

where *r*_f_ is the anisotropy after the cells exposure to polyphenols and *r_i_* the basal anisotropy of the cells.

#### 3.4.2. Transmembrane Potential Evaluation

DiBAC_4_(3) is a slow-response potential-sensitive probe that can enter depolarized cells, binds to the membrane and thus exhibits enhanced fluorescence. Depolarization results in an additional influx of the anionic dye and consequently an increase in the fluorescence. Conversely, hyperpolarization (increase in the absolute value of the membrane potential) is indicated by a decrease in fluorescence [24].

DiBAC_4_(3) labeled PBMCs in a normalized suspension (2 μM probe concentration for 10^5^ cells/mL) were excited at 493 nm and fluorescence emission spectra were collected within the range 500 to 600 nm, with a maximum at 517 nm. Fluorescence intensity was measured before and after 20 min incubation of the cells with Q or EGCG. The membrane potential changes were evaluated as a relative effect of the two polyphenols using Equation (3):

(3) $$e_{V}(\%)=\frac{I_{0}-I_{f}}{I_{0}}\times 100$$

where *I*_0_ is the fluorescence intensity of untreated cells and *I*_f_ is the fluorescence intensity after incubation with Q or EGCG.

The two fluorescent probes were added simultaneously, and the fluorescent signals were collected successively, after performing tests that showed that the two probes do not interfere (discussed above) and the final results were not statistically affected by the successive measurements of the signals.

#### 3.4.3. Statistical Analysis

Results are expressed as means ± standard deviation. For comparison among the groups we used one-way ANOVA and Bonferroni *t*-test, as well as Student’s paired *t* test. Multiple regression analysis was performed to evaluate the interrelations between studied parameters using the Statistical Package for Social Sciences software (SPSS) version 15. For correlations between the parameters, bivariate correlation procedures (Spearman’s correlation coefficent) were used. Differences were considered significant for *p* < 0.05.

## 4. Conclusions

The influence of two natural diet polyphenols (Q and EGCG) on membrane anisotropy and transmembrane potential of PBMCs from normal and hyperglycemic patients was studied, in a double-staining procedure. Q and EGCG displayed a dose dependent hyperpolarizing and stiffening effect on PBMCs, exerted *in vitro*, at physiologic as well as at supraphysiologic levels of exposure.

The PBMCs anisotropy was lower for type 2 diabetes patients compared to normoglycemia ones. Hypercholesterolemia, as well as hyperglycemia strongly impacts the membrane fluidity. Our results show that the increase of the resistin levels and HOMA-IR was associated with a significant increase of the membrane fluidity.

Physiological levels of Q and EGCG tend to normalize the PBMCs membrane anisotropy values of the cells isolated from T2D patients, making it similar to that of PBMCs isolated from normoglycemia ones. The effect of the two tested compounds was low on the more rigid membranes from normoglycemia-normocholesterolemia patients. Both polyphenols tend to increase membrane rigidity, EGCG being more effective than Q. The stiffening effect exhibited by EGCG is greater on cells isolated from hypercholesterolemia patients.

Q and EGCG have a strong hyperpolarizing effect on cells isolated from patients with hypercholesterolemia, regardless of associated hyperglycemia. The hyperpolarizing effect is not linked to the inflammatory profile of the patients. The decrease of the membrane fluidity and the hyperpolarizing effect could explain the cardiovascular protective action of the tested compounds. Flavonoids could improve PBMCs membrane function not only by providing an antioxidant protection through free radical scavenging but also by modulating the membrane fluidity and transmembrane potential.

## Figures and Tables

**Table 1:** Biochemical characterization of the subjects, divided into subgroups depending on their fasting plasma glucose and cholesterol levels.

Biochemical parameters	NGNC (*n* = 15)	NGHC (*n* = 10)	HGNC (*n* = 12)	HGHC (*n* = 14)
**Gli** (mg/dL)	94.95 ± 8.25	90.74 ± 9.11	172.33 ± 82.07 *,^a^	205.48 ± 115.21 *,^a^
**HbA1c** (%)	5.26 ± 0.45	5.80 ± 0.54	7.80 ± 1.67 *,^a^	9.55 ± 0.21 *,^a^
**TC** (mg/dL)	167.29 ± 21.78	226.67 ± 16.72 *,^b^	164.25 ± 22.92	232.91 ± 39.92 *,^b^
**TG** (mg/dL)	92.50 ± 39.92	123.11 ± 74.39	134.17 ± 38.32	146.10 ± 45.56
**HDL** (mg/dL)	43.86 ± 9.69	49.83 ± 6.68	35.63 ± 8.21	52.39 ± 13.51
**LDL** (mg/dL)	104.14 ± 17.73	148.67 ± 11.68 *,^b^	91.60 ± 34.51	142.70 ± 24.21 *,^b^
**AST** (U/L)	24.43 ± 12.15	21.50 ± 4.11	20.90 ± 9.49	23.57 ± 8.67
**ALT** (U/L)	26.71 ± 23.24	20.00 ± 10.72	28.90 ± 14.55	34.71 ± 27.56
**Urea** (mg/dL)	29.62 ± 7.52	31.46 ± 12.82	51.20 ± 29.67	32.80 ± 19.31
**Creatinine** (mg/dL)	0.97 ± 0.21	0.80 ± 0.21	1.33 ± 0.94	0.93 ± 0.24
**Uric acid** (mg/dL)	5.57 ± 1.51	4.06 ± 1.01	5.44 ± 1.59	4.97 ± 1.25

* *p* < 0.05;

a *vs*. NGNC subgroup;

b *vs*. NC matched group (NGHC *vs*. NGNC and HGHC *vs*. HGNC).

Subgroups: NGNC, normogycemia-normocholesterolemia; NGHC, normoglycemia-hyper-cholesterloemia; HGNC, hyperglycemia-normocholesterolemia; HGHC, hypergycemia-hyper-cholesterolemia.

**Table 2:** The inflammatory markers and insulin resistance parameters for NG and HG groups.

Parameter	NG (*n* = 25)	HG (*n* = 26)
**CRP** (mg/L)	1.64 ± 1.47	2.19 ± 2.84
**ICAM-1** (ng/mL)	3.53 ± 1.14	3.77 ± 2.07
**MCP-1** (pg/mL)	156.63 ± 73.66	218.83 ± 107.58 *
**Insulin** (μIU/mL)	11.99 ± 1.87	15.12 ± 10.82
**Resistin** (ng/mL)	103.39 ± 45.15	132.80 ± 29.42 *
**HOMA-IR**	2.80 ± 0.64	6.90 ± 4.71 *

* *p* < 0.05.

**Table 3:** The inflammatory marker values of the subjects divided into subgroups, depending on their fasting plasma glucose and cholesterol levels.

Inflammatory markers	NGNC (*n* = 15)	NGHC (*n* = 10)	HGNC (*n* = 12)	HGHC (*n* = 14)
**CRP** (mg/L)	1.55 ± 1.36	1.84 ± 1.78	1.42 ± 1.38	2.86 ± 3.59
**ICAM-1** (ng/mL)	3.44 ± 1.08	3.66 ± 1.29	3.91 ± 1.84	3.66 ± 2.31
**MCP-1** (pg/mL)	121.11 ± 47.78	206.36 ± 76.54 *	177.27 ± 85.75	254.45 ± 114.35 *

* *p* < 0.05, (each HC subgroup was compared to the matching NC subgroup).

**Table 4:** Insulin resistance parameters for the subjects divided into subgroups, depending on their fasting plasma glucose and cholesterol levels.

Parameter	NGNC (*n* = 15)	NGHC (*n* = 10)	HGNC (*n* = 12)	HGHC (*n* = 14)
**Resistin** (ng/mL)	95.29 ± 47.46	111.50 ± 43.96	128.86 ± 28.49 *	136.03 ± 31.15 *
**HOMA-IR**	2.96 ± 0.73	2.54 ± 0.38	6.53 ± 4.81 *	7.27 ± 4.81 *
**Insulin** (μIU/mL)	12.50 ± 2.20	11.24 ± 0.88	14.38 ± 3.79	15.73 ± 14.54

* *p* < 0.05, compared to NGNC group;

IU: International Units.

## Abbreviations

ALT: alanine aminotransferase
AST: aspartate transaminase
CRP: C-reactive protein
CVD: cardiovascular disease
DMSO: dimethylsulfoxide
EGCG: epigallocatechin gallate
Gli: fasting plasma glucose
HbA1c: glycated haemoglobin
HOMA-IR: homeostatic model analysis for insulin resistance
ICAM-1: intercellular adhesion molecule 1
MCP-1: monocytechemoatractant protein-1
PBMCs: peripheral blood mononuclear cells
Q: quercetin
T2D: type 2 diabetes mellitus
TC: total cholesterol
TG: triglycerides
TMA-DPH: 1-(4-trimethylammonium-phenyl)-6-phenyl-1,3,5-hexatriene *p*-toluensulfonate
DiBAC_4_(3): *bis*-(1,3-dibutylbarbituric acid) trimethineoxonol.
